# Recycled PET Fibers with Dopamine Surface Modification for Enhanced Interlayer Adhesion in 3D Printed Concrete

**DOI:** 10.3390/ma17205126

**Published:** 2024-10-21

**Authors:** Ke-Ke Yu, Tai-Qi Zhao, Qi-Ling Luo, Yang Ping

**Affiliations:** 1 State Key Laboratory of Intelligent Construction and Healthy Operation and Maintenance of Deep Underground Engineering, College of Civil and Transportation Engineering, Shenzhen University, Shenzhen 518060, China; 2110474082@email.szu.edu.cn (K.-K.Y.); 2200471072@email.szu.edu.cn (T.-Q.Z.); 2 Guangdong Provincial Key Laboratory of Durability for Marine Civil Engineering, College of Civil and Transportation Engineering, Shenzhen University, Shenzhen 518060, China; 3 PowerChina Eco-Environmental Group Co., Ltd., Shenzhen 518102, China; pingyang-shj@powerchina.cn

**Keywords:** 3D printed concrete, interlayer adhesion strength, microstructure

## Abstract

Three-dimensional printed concrete (3DPC) is increasingly recognized in the construction industry for its high design flexibility and the elimination of conventional formwork. However, weak interlayer adhesion remains a significant challenge. The potential of recycled polyethylene terephthalate (PET) fibers for reinforcing 3DPC is being explored, driven by their environmental sustainability and economic advantages. However, there is an inadequate interfacial adhesion between these recycled fibers and the 3DPC matrix. This study investigated the use of dopamine modification to address this issue and enhance the interlayer adhesion of fiber-reinforced 3DPC. Recycled PET fibers were surface-modified using dopamine treatment, forming a polydopamine (PDA) film that improved surface roughness and hydrophilicity. Both unmodified and modified fibers were incorporated into 3DPC at various volume fractions (0.1%, 0.3%, 0.5%). The effects on interlayer adhesion strength, compressive strength, and flexural strength were systematically evaluated and compared. The results showed that the inclusion of 0.3 vol% dopamine-modified fibers resulted in a 22.5% increase in interlayer adhesion strength compared to the control group, and a 14.8% improvement over unmodified fibers at the same content. Additionally, the compressive strength and flexural strength of 3DPC with 0.3 vol% MPET fibers increased by 22.5% and 27.6%, respectively, compared to the control group. Microstructural analysis using SEM and XRD revealed that the dopamine modification significantly improved the interfacial adhesion between fibers and the concrete matrix, explaining the superior performance of modified fibers. This study demonstrates that recycled PET fibers modified with dopamine can effectively enhance the interlayer adhesion of 3DPC. The findings affirm that surface modification techniques can significantly elevate the utility of recycled PET fibers in 3DPC, contributing to the sustainable advancement of construction materials.

## 1. Introduction

As global demands for sustainable development and environmental protection continue to grow, the construction industry faces unprecedented challenges in transforming and upgrading. 3DPC technology, with its ability to reduce material waste [1], enables formwork-free construction [2], and utilizes sustainable and recycled materials [3], paving new paths for creating sustainable architectural environments. The potential of 3DPC extends beyond printing small-scale buildings or components, becoming increasingly evident in large-scale construction projects. For instance, 3D printing can be applied in bridges [4], high-rise buildings [5], and even infrastructure projects, where precise control over material use and placement not only enhances structural performance but also facilitates complex design concepts that are often challenging to achieve with traditional construction methods [6].

Despite the broad prospects for 3D printing technology in the construction sector, its practical application faces numerous challenges. Unlike traditionally cast concrete, 3D printed concrete is subject to issues in interlayer regions due to its layer-by-layer extrusion process [7]. Moreover, since the printing and curing processes are exposed to air, 3DPC is more susceptible to durability issues such as shrinkage and cracking [8,9]. These challenges make the material formulation and construction techniques of 3DPC more complex, involving concrete’s compressive and flexural strength, interlayer adhesion strength, and tensile strength. Fibers offer a potential solution, as they can enhance crack resistance and various mechanical properties of concrete when used as a reinforcing material [10,11]. Pham et al. [12] demonstrated significant improvements in the mechanical properties of 3DPC, particularly in flexural strength, by optimizing the length and volume content of steel fibers. Zhang et al. [13] developed a new type of 3D printing extruder that manufactures continuous carbon fiber-reinforced polymer (CCFRP) composites using an in situ impregnation technique, significantly enhancing the composite’s flexural strength and eliminating performance discrepancies between traditional and 3D printed concrete. Meanwhile, Zhang et al. [14] developed an engineered cement composite (ECC) that combines polyvinyl alcohol (PVA) fibers and activated carbon powder (ACP) for 3DPC, discovering that, while PVA fibers decrease the workability and compressive strength, the addition of ACP enhances both compressive and flexural strength. Although traditional fibers like steel, carbon, and PVA can improve concrete’s mechanical properties, each has limitations, such as the high cost of steel and carbon fibers, particularly carbon fibers, which restricts their use in large-scale construction projects. Upon water contact, PVA fibers may swell, potentially affecting the overall performance and durability of concrete [15]. In the search for more economical and environmentally friendly fiber materials, PET fibers are emerging as a focus of research due to their low cost and recyclability.

Although PET fibers have been used to enhance traditional concrete, improving its crack resistance and impact strength, their application in 3DPC is relatively limited. Current research primarily focuses on the use of unmodified fibers [16,17,18], which often limits performance enhancements due to poor compatibility with concrete. Borg et al. [16] indicated that fibers made from waste PET plastic can effectively strengthen concrete, despite slightly reducing its compressive strength, significantly enhancing crack resistance and toughness, particularly with 1.5% volume of 50 mm long deformed fibers, reducing crack width by 68.7%. Tang et al. [17] showed that recycled foam concrete reinforced with recycled PET fibers not only performs better in dry density and compressive strength compared to ordinary PET fiber-reinforced recycled foam concrete, but also exhibits improved frost resistance and chemical corrosion resistance. Therefore, it is necessary to optimize the interface adhesion between fibers and the 3DPC matrix through surface modification techniques to further enhance structural performance. Common modification techniques for PET include plasma treatment [19], silane coupling agent treatment [20], graphene oxide or reduced graphene oxide treatment [21,22], dopamine treatment [23], and alkali treatment [24]. Compared with other surface treatment methods, dopamine modification has significant advantages. While plasma treatment can effectively modify the surface of PET fibers, it requires specialized equipment and strict process control, resulting in high costs [19]. Silane coupling agent treatment can improve interfacial adhesion, but the process is complex and poses certain environmental and health risks [20]. The preparation of graphene oxide coatings is relatively time-consuming, and the reduction process involves the use of chemical reagents, such as sodium hydroxide, which pose potential pollution risks [21]. Although alkali treatment is simple to operate, it can lead to a decline in the mechanical properties of the treated PET fibers [24]. In contrast, dopamine modification is easy to perform. It can spontaneously deposit on the surface of PET fibers under mild conditions without the need for complex equipment. A preliminary cost analysis shows that the modification of 1 kg of recycled PET fibers costs approximately CNY 610. This includes CNY 300 for the recycled PET fibers, CNY 150 for dopamine, CNY 150 for the Tris-HCl buffer solution, and an estimated CNY 10 for other materials and energy costs. While this represents a significant increase from the cost of unmodified recycled PET fibers, the improved performance of the modified fibers could potentially lead to reduced overall material requirements in 3D printed structures, possibly offsetting the initial cost increase.

This study, for the first time, explores the enhancement of interlayer adhesion in 3DPC through the application of dopamine-modified recycled PET fibers, while also investigating the effects on compressive strength and flexural resistance. Initially, the surface modifications of the recycled PET fibers were characterized using scanning electron microscopy (SEM), water contact angle measurements, and X-ray photoelectron spectroscopy (XPS). Subsequently, a comprehensive set of tests was conducted to evaluate the impact of these dopamine-modified fibers on 3DPC performance. The primary focus of this study was interlayer adhesion evaluations, which were included in these tests along with assessments of compressive strength and flexural resistance. The study concludes by elucidating the mechanisms underlying the fibers’ reinforcement effects, particularly on interlayer adhesion, through X-ray diffraction (XRD), and further SEM analyses. This research highlights the potential of dopamine-modified recycled PET fibers to significantly enhance the mechanical properties and sustainability of 3DPC, offering a promising approach for eco-friendly construction practices.

## 2. Materials and Methods

### 2.1. Materials

**Figure 1:**
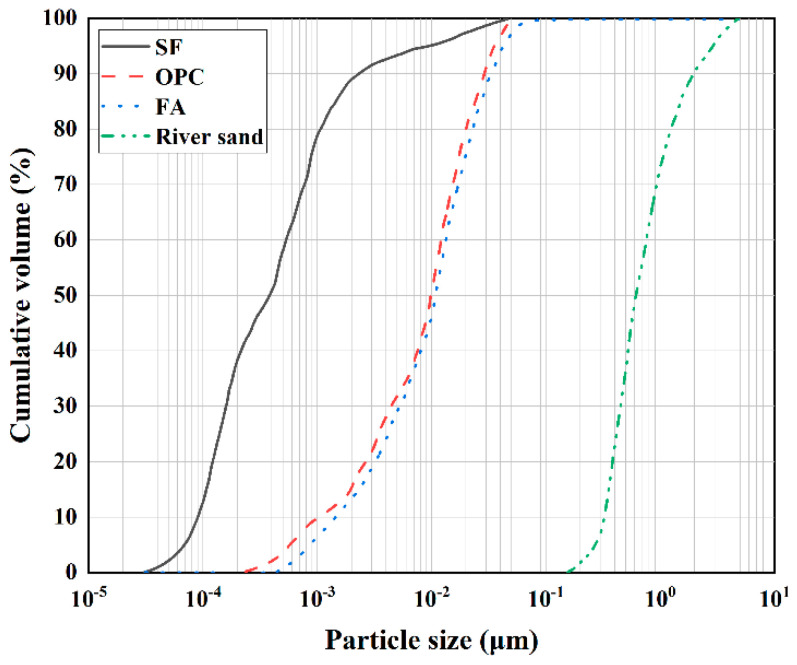
Particle size distribution of cementitious materials and river sands.

This study used ordinary Portland cement (OPC), fly ash (FA), and silica fume (SF) as the cementing materials to prepare the matrix for 3DPC. The ordinary Portland cement used was P·Ⅰ national standard cement with a strength grade of 52.5. The fly ash was model V1500-I, and the silica fume was model V2000-95D. OPC, FA, and SF were sourced from Wuhan Weishen Technology Development Co., Ltd. (Wuhan, China), and their chemical compositions are shown in Table 1. The fine aggregate used was river sand, produced by Xiamen ISO Co., Ltd. (Xiamen, China). The particle size distribution of the cementing materials and aggregates is shown in Figure 1. To improve the fluidity of the fresh concrete and make it suitable for 3D concrete printer extrusion, this study used a polycarboxylate superplasticizer (SP) produced by Beijing Shichuang Lianhe (Beijing, China), with a water reduction rate of over 32%.

Recycled PET chopped fibers, with an average length of 6 mm, a density of 1.38 g/cm³, and an average diameter of 18 μm, were used, purchased from Shandong Yitai Engineering Materials Co., Ltd. (Tai’an, China). The surface modification materials included dopamine hydrochloride, tris (hydroxymethyl) aminomethane hydrochloride (Tris-HCl) buffer solution, and deionized water. Dopamine hydrochloride, with a molecular weight of 189.64 and appearing as a white crystalline powder, was purchased from Shanghai Aladdin Chemical Reagent Co., Ltd. (Shanghai, China). The Tris-HCl buffer solution, with a concentration of 1 mol/L and a pH of 8.8, was purchased from Macklin Reagent Company (Shanghai, China).

### 2.2. Surface Modification of Recycled PET Fibers

**Figure 2:**
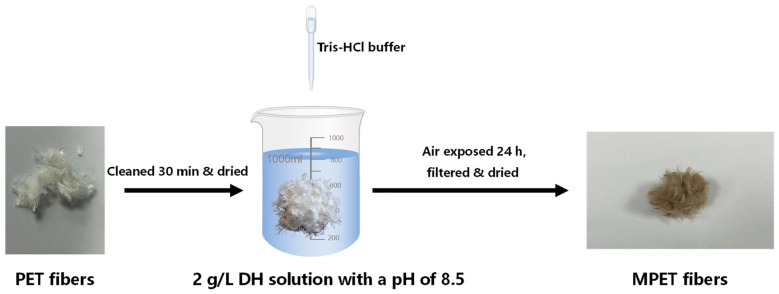
Recycled PET fibers modification process.

Figure 2 shows the modification process of recycled PET fibers. The PET fibers were ultrasonically cleaned in deionized water for 30 min and then dried to a constant weight in an oven at 60 °C. A 1.6 g sample of dopamine powder was dissolved in 800 mL of deionized water, and the pH was adjusted to 8.5 using a Tris-HCl buffer solution, resulting in a modification solution with a concentration of 2 g/L. Fifteen grams of dried PET fibers were then placed in the modification solution, thoroughly stirred with a glass rod, and left exposed to air for 24 h. The modification process was carried out at room temperature (25 °C). The mixture was stirred intermittently with a glass rod every 2 h during the 24 h period. After modification, the fibers were filtered using filter paper and rinsed thoroughly with deionized water to remove any unreacted dopamine. The modified PET (MPET) fibers were obtained by filtering the solution and drying the fibers to a constant weight in an oven at 60 °C.

### 2.3. Mix Design and Preparation Methods of 3D Printed Concrete

To study the effect of the surface modification of fibers with different dosages on 3DPC, 3DPC samples with various mix proportions were prepared. Table 2 presents the mix proportions of 3DPC with different volume fractions of fibers (0%, 0.1%, 0.3%, 0.5%). Cubic specimens (40 × 40 × 40 mm) and prismatic specimens (40 × 40 × 160 mm) were cast using standard molds for experimental purposes. These cast specimens were used to evaluate the basic mechanical properties of the mixtures, such as compressive and flexural strength. The casting process involved pouring the freshly mixed concrete into the molds, followed by vibration to ensure proper compaction and to eliminate air voids. The concrete was prepared using a handheld mixer (with a maximum motor power of 3700 W).

Initially, the required dry concrete materials were placed in the mixing bowl according to the mix proportions and stirred at low speed for 1.5 min. Subsequently, a mixture of water and SP was added and stirred at low speed for 1 min, followed by high-speed stirring for 1 min. Mixing was paused for 1 min, during which unmixed areas were transferred to the mixing zone with a spatula. Finally, the mixture was stirred rapidly for 2 min. The 3D printing was performed using a custom-built concrete printer with a 20 mm diameter nozzle, operating at a speed of 1 rotation per second, depositing 14 layers to create cuboid specimens of 150 mm × 30 mm × 10 mm dimensions. The cast and printed specimens were maintained in a controlled indoor environment with a temperature of 25 °C and a relative humidity of 95%, until they reached the specified age.

### 2.4. Compressive and Flexural Strength Tests

Compression and flexural strength tests were conducted on specimens at 28 d of curing. Six samples were tested for each experimental group. The tests for the compressive and flexural strength of concrete were performed according to the national standard GB/T17671-2021 [25]. The compressive strength and flexural strength were measured using cubic specimens with dimensions of 40 × 40 × 40 mm and rectangular specimens with dimensions of 40 × 40 × 160 mm, respectively. The compressive and flexural strength tests were conducted using a computer-controlled electronic universal testing machine (YAW-300b) with loading rates of 2.4 kN/s and 50 N/s.

### 2.5. Interlayer Adhesion Strength Tests

**Figure 3:**
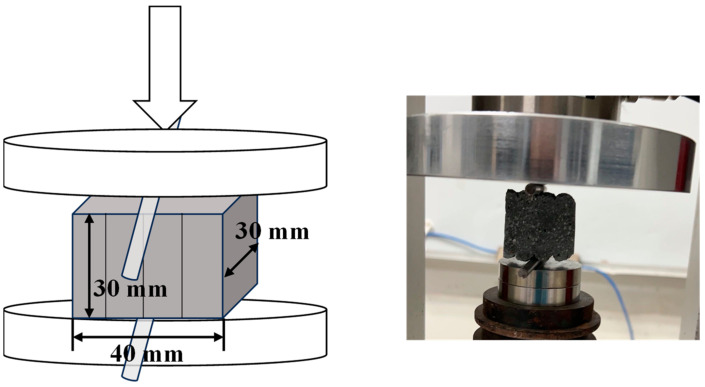
Split tensile strength test.

In this study, the interlayer split strength testing method recommended by T/CECS786-2020 was employed to directly test the interlayer split strength of 3DPC [26]. A cutting machine was used to cut the cured 14-layer 3DPC specimens into split test samples of 30 × 30 × 40 mm. Samples from layers 4 to 7 and 10 to 13 of each mix proportion were selected for split tests, with no fewer than 6 samples per group. The test results were averaged arithmetically to obtain the final values. Figure 3 is a schematic diagram of the split strength test for 3DPC. Due to the uneven sides of the printed samples and the depressions between the printing layers, steel rods of 5 mm diameter were placed between the platen and the split test samples, positioned at the interlayer locations, with both rods placed at the same depression of the printing layers [27].

### 2.6. Microstructural Characterization, Chemical Analysis, and Fiber Hydrophilicity Tests

In this study, the Quanta TM250FEG field emission scanning electron microscope (SEM) was employed to examine the fibers and their distribution in 3DPC, focusing on the surface micro-morphology, distribution within the concrete, interface characteristics with the matrix, and micro-cracks, among other structural details. Prior to scanning, specimens were extracted from damaged interlayer strength test samples and subjected to polishing and grinding. Additionally, the chemical composition of the fibers was analyzed using the PHI5000 Versaprobe III X-ray photoelectron spectrometer (XPS) from ULVAC-PHI, Inc. (Chigasaki, Japan). Further, the X’ Pert series X-ray diffractometer (XRD) from Malvern Panalytical, with Cu-Kα radiation, was used to explore the crystalline composition of the concrete hydration products. Diffraction data were collected at a scanning speed of 6°/min, covering a range of 10° to 70°, and detailed analysis was performed on the diffraction spectrum. Finally, the hydrophilicity of the fibers was assessed using a surface tensiometer from Dataphysics, Germany. The fibers were compressed using a press; deionized water was applied to the flattened fibers, and the water contact angle was measured.

## 3. Experimental Results and Analysis

### 3.1. Properties of Recycled PET and Its Modified Fibers

#### 3.1.1. Apparent Morphology of the Fibers

**Figure 4:**
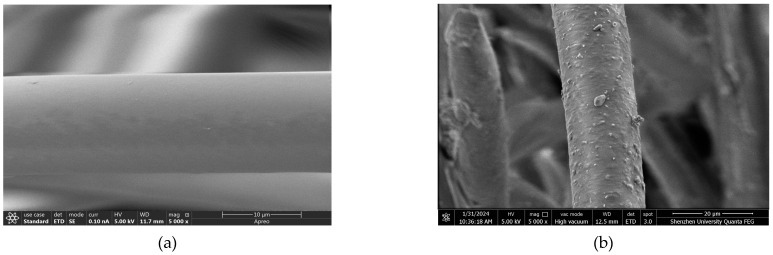
SEM images of (**a**) PET; (**b**) MPET.

Figure 4 displays the microscopic morphological transformations of recycled PET fibers before and after undergoing dopamine treatment. Initially, the unmodified fibers (Figure 4a) present a relatively smooth exterior. Following a 24 h treatment with dopamine, the fibers exhibit numerous prominent protrusions, markedly enhancing their surface roughness (Figure 4b). These alterations indicate the effective role of dopamine in increasing fiber roughness. According to previous studies, dopamine undergoes oxidation and spontaneously self-polymerizes in an alkaline environment with the presence of oxygen, forming a polydopamine (PDA) coating on the surfaces of PET fibers [28,29].

#### 3.1.2. Hydrophilicity Analysis

**Figure 5:**
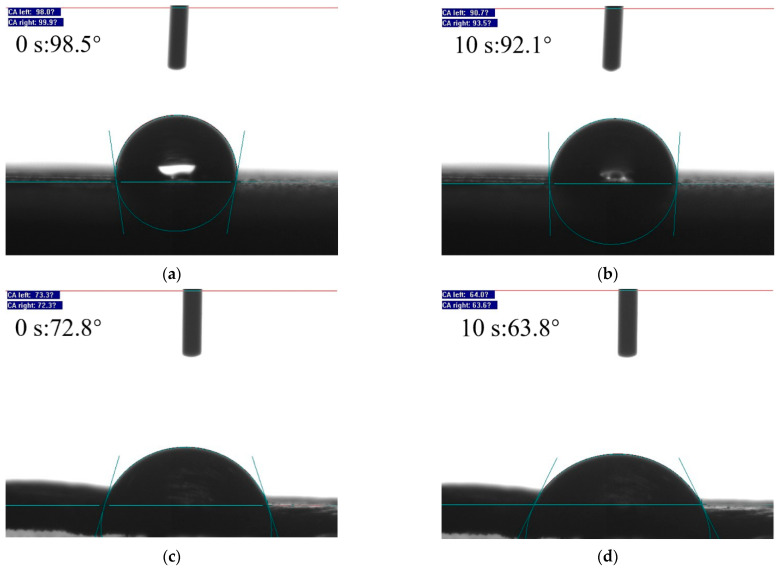
Water contact angles: (**a**,**b**) PET; (**c**,**d**) MPET.

Initially, the PET fibers displayed a water contact angle of 98.5° at 0 s (Figure 5a), indicative of their hydrophobic nature. Post-modification, the MPET fibers showed a reduction in contact angle to 72.8° (Figure 5c), thereby improving their hydrophilicity. To further assess the stability of this alteration, the water contact angles on the fiber surfaces were measured after a 10 s exposure. The data revealed a modest decrease in the contact angle for the PET fibers, from 99° down to 92.1° (Figure 5b), while the MPET fibers experienced a more substantial reduction from 72.8° to 63.8° (Figure 5d). These findings underscore the increase in hydrophilicity of the MPET fibers compared to the PET fibers. This is primarily attributed to the hydroxyl and amine groups in PDA [23].

#### 3.1.3. XPS Analysis

**Figure 6:**
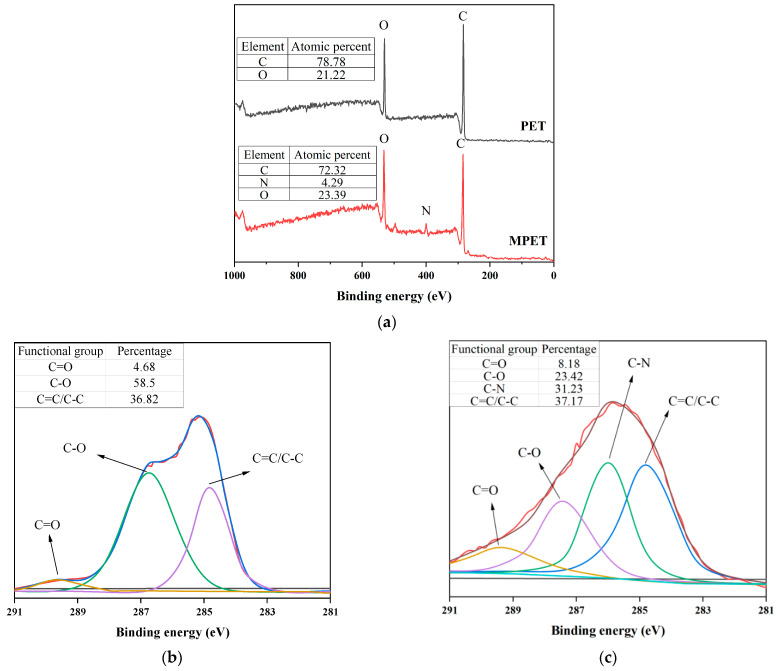
XPS spectra of PET and MPET fibers: (**a**) broad spectra; (**b**) C1s spectra of PET; (**c**) C1s spectra of MPET.

The dopamine modification of PET fibers was investigated to determine changes in their surface chemical structures through X-ray Photoelectron Spectroscopy (XPS), as illustrated in Figure 6. Broad spectrum analysis (Figure 6a) indicated that unmodified PET fibers predominantly contained carbon (C) and oxygen (O) elements. Post-modification, a new nitrogen (N) peak was observed in the MPET fibers, validating the successful deposition of the PDA layer on the fibers [21].

Figure 6b and Figure 6c depict the C1s spectra of PET and MPET fibers, respectively. Notably, the relative content of C=O at approximately 289 eV in MPET fibers increased from 4.68% to 8.18%. Conversely, the relative content of C-O functional groups at 286.8 eV decreased from 58.5% to 23.42% in MPET fibers, suggesting that the PDA coating formed by the polymerization of dopamine molecules may obscure portions of the PET surface [30]. Additionally, a novel C-N peak near 285.6 eV, constituting 31.23% of the relative content, was noted in the MPET fibers’ C1s spectra, signifying the formation of amide bonds through reactions between dopamine’s amino groups and the carbonyl groups on the PET fiber surface [31,32]. The stability of the C=C/C-C peaks at 284.8 eV before and after modification confirm that the core carbon chain structure of the PET fibers was preserved during the modification process. Overall, dopamine modification modified the surface chemical structure of PET fibers. The introduction of nitrogen elements and the increase in C-N functional groups enhanced the polarity of the PET fibers, thereby improving their interface interactions with the concrete matrix [33].

### 3.2. Mechanical Properties of Fiber-Reinforced 3DPC

#### 3.2.1. Compressive Strength

**Figure 7:**
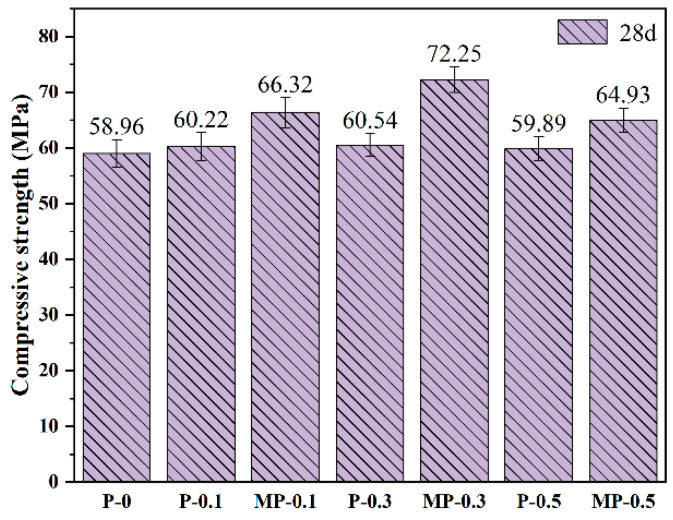
Compressive strength of PET and MPET fiber concrete. Note: The numbers above each bar indicate the average compressive strength, and error bars represent the standard deviation, illustrating variability in the results.

Figure 7 illustrates the variations in the compressive strength of concrete specimens reinforced with PET and MPET fibers at different dosages (0.1 vol%, 0.3 vol%, and 0.5 vol%) over curing periods of 28 d. The improvements were not significant at 28 d, indicating that PET fibers have a limited effect on the compressive strength of concrete. In contrast, specimens containing MPET fibers showed a significant increase in compressive strength at all tested curing periods, especially after 28 d, where the MP-0.3 group attained a peak strength of 72.25 MPa, 22.5% higher than the control group (P-0). This significant increase is likely attributable to the presence of polar groups on the modified fibers, which intensified their interactions with the concrete matrix. The increased surface roughness of the fibers further improved their mechanical interlocking with the concrete matrix [34,35]. Additionally, the study demonstrated that when the fiber dosage was increased to 0.5 vol%, compressive strength did not improve for either PET or MPET fibers; in fact, it was slightly reduced compared to the 0.1 vol% level, presumably due to fiber aggregation or uneven distribution beyond the optimal dosage, consequently diminishing the adhesion strength with the concrete matrix and negatively impacting the material’s mechanical properties [36,37].

#### 3.2.2. Flexural Strength

**Figure 8:**
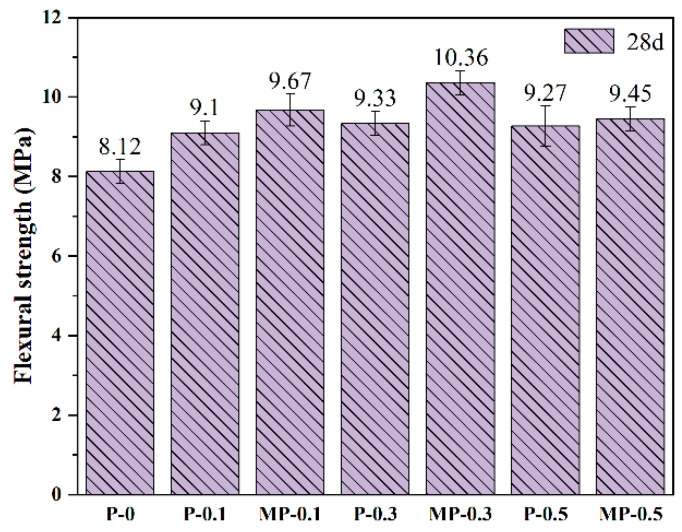
Flexural strength of PET and MPET fiber concrete. Note: The numbers above each bar indicate the average flexural strength, and error bars represent the standard deviation, illustrating variability in the results.

Figure 8 shows the changes in flexural strength of concrete specimens after 28 d of curing. The specimens in the MP-0.3 group exhibited significant improvements, recording an 11% increase in strength over the P-0.3 group and a 27.6% increase over the P-0 group; these were the highest enhancements observed among all specimens. These results suggest that modified fibers significantly bolster the long-term load-bearing capacity of the concrete. This is attributed to the bridging effect of the modified fibers [38]. However, when the fiber dosage was increased to 0.5 vol%, the growth in flexural strength slowed down or even decreased for all specimens. Specifically, at 28 d, the flexural strength of the MP-0.5 group was 9% lower than that of the MP-0.3 group. A similar trend was observed with PET fibers, indicating that excessive fiber content may undermine interfacial adhesion strength, likely due to fiber aggregation and uneven distribution [36,37].

#### 3.2.3. Interlayer Adhesion Strength

**Figure 9:**
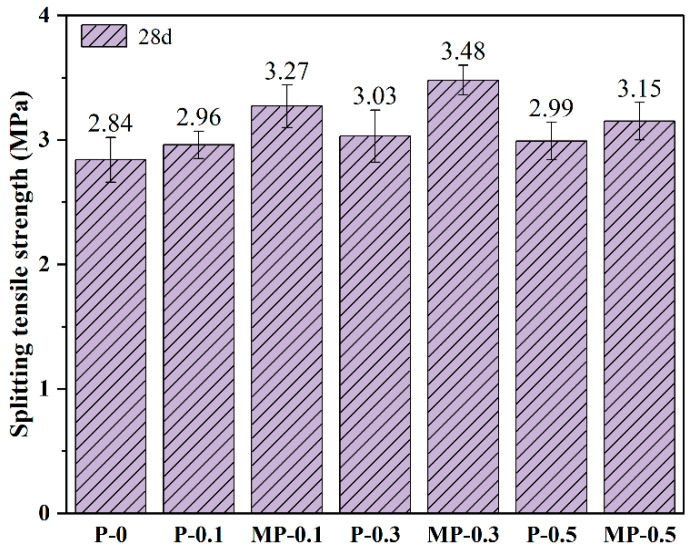
Interlayer adhesion strength of 3DPC.

Figure 9 analyzes the effects of different dosages of PET and MPET fibers on the interlayer adhesion strength in 3DPC, based on 28 d split tensile strength test results. The data demonstrated that all specimens containing PET and MPET fibers exhibited an improvement in interlayer adhesion strength compared to the control group P-0. Specifically, the split tensile strength of the P-0.1 group, with a 0.1 vol% dosage of PET fibers, increased by 4.2%, while the MP-0.1 group with the same dosage of MPET fibers saw a significant improvement of 15.1%. This indicated that even a small addition of fibers can notably enhance the interlayer adhesion performance of 3DPC, and surface modification can further amplify this effect. This is due to the presence of fibers at the interlayer interfaces and within the printed layers of 3DPC, which connect the upper and lower layers of the printed specimens, thereby enhancing the interlayer adhesion of 3DPC [39]. When the fiber content was increased to 0.3 vol%, the split tensile strength for PET fibers rose to 3.03 MPa, and the MPET fibers reached a peak strength of 3.48 MPa, demonstrating the substantial impact of modified fibers on optimizing interface adhesion. This is primarily attributed to the bridging effect of the modified fibers [38,40]. Meanwhile, the PDA layer increased the fiber’s surface roughness and hydrophilicity, thereby enhancing the interfacial adhesion between the fibers and the concrete matrix [23]. However, at a 0.5 vol% fiber content, the split tensile strength of the unmodified fibers slightly decreased to 2.99 MPa, potentially due to fiber aggregation creating weak points [39]. Despite a decrease to 3.15 MPa, the MPET fibers remained superior to the PET fibers. Overall, the incorporation of fibers significantly improved the interlayer adhesion strength of 3DPC, particularly the surface-modified MPET fibers, which showed a more pronounced effect.

### 3.3. Analysis of Interlayer Adhesion Mechanism

#### 3.3.1. XRD Analysis

**Figure 10:**
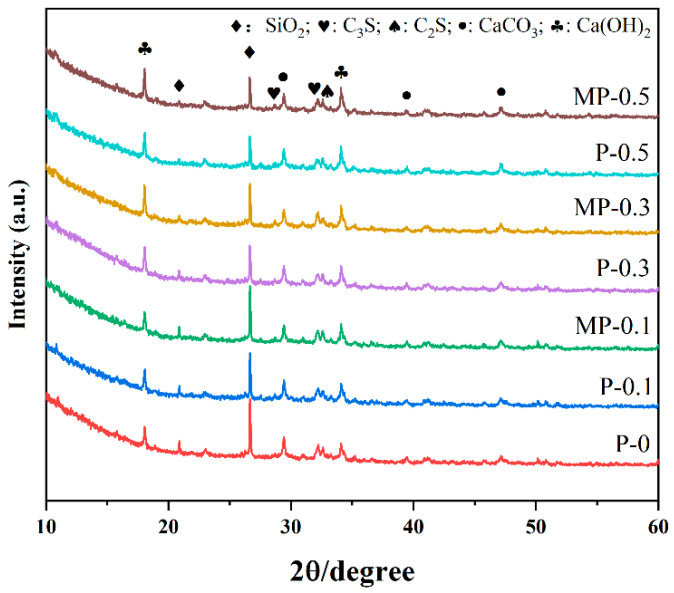
The XRD pattern of 3DPC.

From Figure 10, it can be observed that the hydration products in the 3DPC samples primarily included C-S-H gel, calcium hydroxide (CH), calcium carbonate, SiO_2_, and unhydrated C_3_S and C_2_S. Regardless of whether it was the control group or groups with added fibers or modified fibers, the SiO_2_ peak was prominent after 28 d of curing, due to the inclusion of sand containing SiO_2_ in the concrete. Apart from the SiO_2_ peak, no significant changes were observed in the diffraction peaks of other hydration products, further confirming that the addition of fibers had a limited impact on the main hydration processes [23].

#### 3.3.2. SEM Analysis

**Figure 11:**
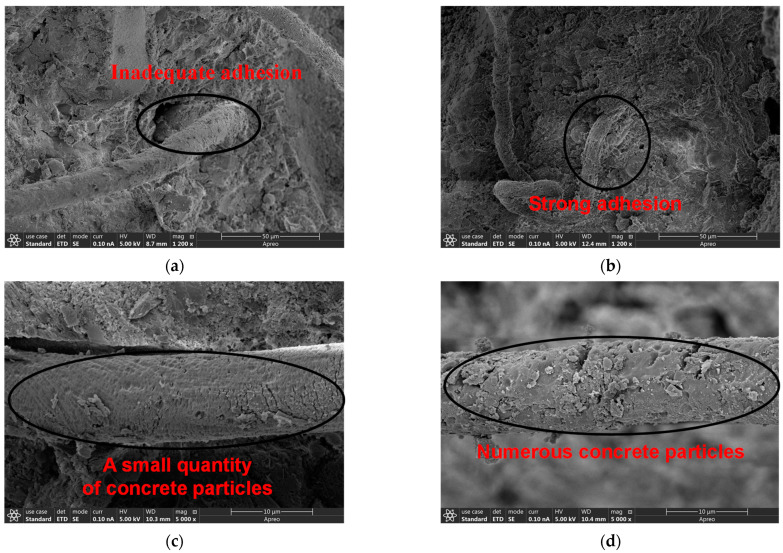

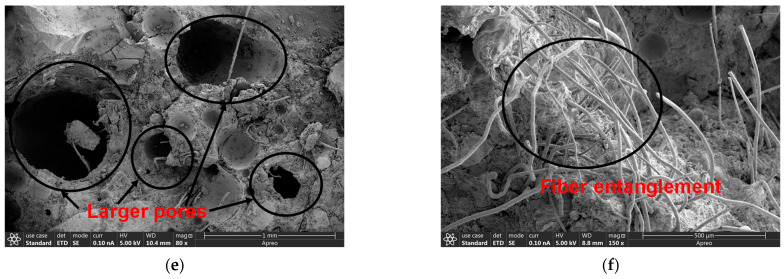
The SEM of 3DPC: (**a**) PET fiber-concrete interface; (**b**) MPET fiber-concrete interface; (**c**) PET fiber-concrete pullout interface; (**d**) MPET fiber-concrete pullout interface; (**e**) pores in the P-0.5; (**f**) fiber distribution in the P-0.5.

Figure 11 presents SEM images that examine the cross-sections of 3DPC after the interlayer adhesion strength test. The images analyze the interface adhesion between different types of fibers and the concrete matrix. As shown in Figure 11a, significant gaps existed between the PET fibers and the concrete, indicating unstable interface adhesion. In contrast, Figure 11b illustrates that the MPET fibers are well-bonded to the concrete, suggesting that these modified fibers significantly enhance interface adhesion. The modified fibers have many hydrophilic groups, such as amino groups on their surface, which can enhance their adhesion with concrete [23]. This finding corroborates the enhancement in mechanical strength discussed in Section 3.2. This improvement is crucial for enhancing the interlayer adhesion strength and overall mechanical properties of 3DPC.

Observations of fiber pull-out interfaces, as shown in Figure 11c, reveal that PET fibers pulled out with only a few concrete particles adhering, whereas the rough-surfaced MPET fibers, shown in Figure 11d, retained more concrete particles, suggesting a tighter bond with the cement matrix [16,23]. Figure 11e,f show that at a fiber content of 0.5 vol%, the concrete contained larger pores and complex microstructures due to fiber entanglement, which may reduce 3DPC uniformity and create potential weak points under stress concentration, thereby diminishing its mechanical performance. Fiber aggregation was observed to increase with higher fiber content, particularly at 0.5 vol%. This aggregation can be attributed to the high aspect ratio of the fibers and their tendency to entangle [39]. However, at higher concentrations, the increased number of fiber–fiber interactions still led to some degree of aggregation. This further confirms the decrease in mechanical properties at a fiber content of 0.5 vol% discussed in Section 3.2.

Overall, the SEM analysis confirmed the effectiveness of fiber-reinforced concrete, particularly the advantages of modified fibers in enhancing interface adhesion. However, excessive fiber content could lead to adverse microstructural changes that impact the mechanical strength of the 3DPC.

## 4. Mechanism Analysis

By modifying recycled PET fibers, a significant enhancement in the interlayer adhesion of 3DPC was achieved, especially when the MPET fiber content was at 0.3 vol%. Experimental observations indicated that a fiber volume of 0.5 vol% can lead to fiber entanglement and knotting. Modified PET fibers exhibited increased roughness and hydrophilicity on their surfaces, along with various polar functional groups, all of which contributed to improved adhesion and interlocking with the cement matrix. The amine (-NH_2_) and hydroxyl (-OH) groups can form hydrogen bonds with silanol (Si-OH) groups in C-S-H gel, undergo electrostatic interactions with silicate species, and create complexes with calcium ions from CH and C-S-H, thereby enhancing the chemical bonding between the fibers and the cement matrix [41,42].

**Figure 12:**
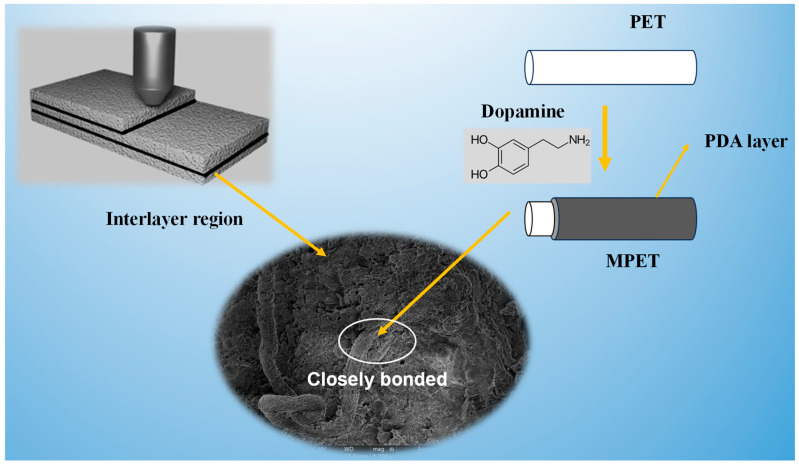
Schematic diagram of the enhancement mechanism of 3DPC mechanical properties.

As illustrated in Figure 12, during the modification process, dopamine underwent oxidation reactions in an alkaline environment through its phenolic hydroxyl and amine groups, self-polymerizing to form a PDA film on the recycled PET fiber surfaces [28,29]. This PDA layer increased the fibers surface roughness and hydrophilicity, thereby enhancing the interfacial adhesion between the fibers and the concrete matrix [23,34,35]. The presence of polar and functional groups (such as amino and phenolic hydroxyl groups) on the PDA surface aided in dispersing the recycled PET fibers within the cement slurry, preventing aggregation to some extent and thus more effectively utilizing the fiber-reinforced concrete properties [43]. Moreover, the PDA film formed a denser transition zone around the fibers, reducing porosity and defects, and thus enhancing the overall performance of 3DPC [44]. When subjected to external loads, the bridging effect of the modified fibers effectively increased energy dissipation, further enhancing the interlayer adhesion of 3DPC [38].

## 5. Conclusions

This research, for the first time, investigated the efficacy of dopamine-modified recycled PET fibers in enhancing the mechanical properties of 3DPC. The study encompassed detailed analyses of fiber morphology, hydrophilicity, and chemical composition, as well as their impact on the mechanical characteristics of 3DPC. This study demonstrates the potential of chemically modified recycled fibers to enhance the performance of 3DPC construction materials. The major conclusions are as follows:

1. Through SEM, water contact angle, and XPS tests, it was demonstrated that by modifying recycled PET fibers with dopamine, a significant enhancement in fiber surface roughness and hydrophilicity was achieved. The modification also increased the presence of nitrogen elements and C-N functional groups on the fiber surfaces.
2. At 28 d, the compressive strength, flexural strength, and interlayer adhesion strength of 0.3 vol% MPET fibers were increased by 19.3%, 11%, and 14.9%, respectively, compared to the same volume of recycled PET fibers. Increasing fiber content to 0.5 vol% did not yield further improvements and in some cases led to slight decreases in performance, likely due to fiber aggregation.
3. According to the results of XRD and SEM analyses, the fibers and modified fibers do not affect the hydration process of 3DPC, the PDA layer ensures tight bonding between the modified fibers and the matrix. This confirms that the mechanical enhancement of 3DPC is primarily attributed to the bridging effect and improved interfacial bonding between the modified fibers and concrete matrix, revealing the mechanisms behind the enhancement of both interlayer adhesion and overall mechanical strength.

This study showed that using recycled PET fibers, modified with an environmentally friendly dopamine technique, can enhance the performance of 3DPC. This has implications for sustainable developments in the building materials industry. The technique not only contributes to the eco-friendliness and cost-effectiveness of construction materials but also offers a novel approach to recycling discarded plastics. In future research, it is necessary to explore the long-term durability of such fibers in concrete, providing insights for advancing sustainable construction practices.

## Figures and Tables

**Table 1:** Chemical compositions of OPC, FA and SF (%).

	CaO	SiO_2_	Al_2_O_3_	MgO	K_2_O	Na_2_O	Fe_2_O_3_	TiO_2_	SO_3_	LOI
OPC	67.00	18.62	5.41	1.30	0.46	0.30	2.98	0.36	3.10	-
FA	6.69	52.45	29.35	0.83	1.07	0.89	5.93	1.24	0.74	4.41
SF	0.16	99.23	0.32	0.12	0.15	-	-	-	0.62	3.47

**Table 2:** Mix proportions of 3DPC.

Gruop	Water (g)	OPC (g)	FA (g)	SF (g)	River Sand (g)	SP (g)	PET (vol%)	MPET (vol%)
P-0	270	630	180	90	1350	3.42	0	0
P-0.1	270	630	180	90	1350	3.42	0.1	0
MP-0.1	270	630	180	90	1350	3.42	0	0.1
P-0.3	270	630	180	90	1350	3.42	0.3	0
MP-0.3	270	630	180	90	1350	3.42	0	0.3
P-0.5	270	630	180	90	1350	3.42	0.5	0
MP-0.5	270	630	180	90	1350	3.42	0	0.5

## Data Availability

The original contributions presented in the study are included in the article, further inquiries can be directed to the corresponding author/s.
